# Optimizing metal part distortion in the material extrusion-thermal debinding-sintering process: An experimental and numerical study

**DOI:** 10.1016/j.heliyon.2024.e28899

**Published:** 2024-04-02

**Authors:** Xueying Wei, Xujun Li, Rüdiger Bähr

**Affiliations:** a Institute of Manufacturing Technology and Quality Management, Otto-von-Guericke-University Magdeburg, Magdeburg, 39106, Germany; b State Key Laboratory of Multiphase Flow in Power Engineering, Xi'an Jiaotong University, 710049, Xi'an, China

**Keywords:** Additive manufacturing, Material extrusion, Thermal debinding, Metal parts, Distortion, Porosity, Computational fluid dynamics

## Abstract

The thermal debinding-sintering process plays an essential role in the context of material extrusion-based additive manufacturing (AM) for producing parts using metal injection molding (MIM). During thermal debinding, metal parts often experience material distortion and porosity, which negatively impacts their mechanical properties. Slowing down the debinding speed is a common approach to mitigate material distortion and porosity. However, this leads to a significant increase in the debinding time. In this study, we carried out debinding-sintering experiments to optimize the distortion and porosity in metal parts. These metal parts were manufactured utilizing bronze/polylactide (PLA) blend filaments and placed in crucibles of different sizes (small, medium, and large), with different heating rates and holding times. The results revealed that the small crucible yielded higher porosity levels in the metal parts, which could be reduced from 23% to 12% by extending both the heating and holding times. In contrast, the medium crucible managed to reduce porosity to approximately 15% without requiring an extension of the processing time. The large crucible, on the other hand, couldn't achieve further porosity reduction due to challenges in reaching the desired temperature. To gain a deeper insight into temperature distribution during the debinding process, we performed numerical simulations using the computational fluid dynamics (CFD) technique and obtained temperature profiles within the kiln using the three crucibles. Ultimately, we carried out standard mechanical tests on the resulting metal parts and evaluated the thermal debinding procedure under various conditions. The approach we employed, combining experiments and numerical simulations, demonstrated significant promise for enhancing the quality of metal parts in the thermal debinding-sintering process.

## 1 Introduction

Metal injection molding (MIM) is one of the most common processes for metal production and has been widely applied in mechanical engineering and the automotive industry [[1], [2], [3], [4], [5]]. Nonetheless, the process has some significant limitations, particularly when it comes to manufacturing metal parts in small batches or with complex geometries. Fortunately, additive manufacturing (AM) has emerged as an effective solution to these issues, particularly with the rise of material extrusion (MEX) and metal/polymer hybrid filaments. Not only does AM offer the advantages of small-batch production and fabrication of complex structures, but it also delivers economic and environmental benefits [6]. Consequently, researchers have shown great interest in utilizing metal/polymer blend filaments in AM due to the exceptional properties of the resulting products [7,8].

Catalytic debinding, solvent debinding, and thermal debinding are three commonly employed debinding methods in the industry. In catalytic debinding, the binder polyacetal is transformed into formaldehyde in the presence of nitric acid, facilitating the conversion of the binder from a solid to a gaseous phase and leaving behind green parts [[8], [9], [10]]. However, the need for a specific debinding machine can significantly increase costs. Solvent debinding, on the other hand, involves the removal of the first part of the binders using a chemical solvent such as acetone or ethanol. The second part of the binder, commonly referred to as a backbone, supports the brown parts and is vaporized under high temperatures at the beginning of sintering [9,[11], [12], [13]]. Thermal debinding entails placing the green parts in refractory ballast, which is stable under high temperature and pressure, to maintain the geometry of the parts during the debinding and sintering processes. Unlike catalytic and solvent debinding methods, the thermal process does not require gaseous nitric acid or chemical solvents, with only a kiln and refractory ballasts necessary for the process [4,9,[14], [15], [16], [17]], making it an environmentally friendly and cost-effective method for producing metal parts via MEX. Nevertheless, incomplete or unsuccessful debinding can cause distortion or porosity in metal parts, leading to reduced density and tensile stress, as well as changes in shrinkage and surface properties [[9], [10], [11],^18^,^19^]. Consequently, present challenges in thermal debinding include eliminating the binder from the green parts completely and minimizing reaction time [15].

In recent decades, many researchers have studied the distortion or porosity of metal parts produced during the metal/polymer filament and MEX process. Thompson et al. [19] reported a better metal surface by reducing the debinding heating rate from 3 K/min to 0.2 K/min. Moreover, increasing the sintering temperature to 20 K could improve the porosity inside the metal parts. In our previous work [4], we discovered that the location of distortions depends on the printing direction. Due to the compact combination of infill, distortions easily appeared on the surface of the metal part parallel to the build platform. This distortion could be reduced by controlling the printing orientation. Gonzalez-Gutierrez et al. [11] determined the debinding level that influences distortion. They found that incomplete removal of binder forms pores on the metal parts' surface. Songh et al. [20] used a two-step debinding process to reduce the thermal debinding time effectively. Supriadi et al. [16] evaluated the effects of the holding time, debinding temperature, and heating rate of the thermal debinding process on the density and porosity of the final parts. A longer holding time, higher debinding temperature, and lower heating rate were found to produce a higher density and smaller pores for the metal parts. Tafti et al. [3] found that a low pre-sintering temperature enhanced the microstructure of the interior of the metal parts. Using a sintering holding time of 3 h significantly increased the density of the metal parts [2]. Ravi et al. [18] explored solutions for minimizing the distortion of the final metal parts, such as using a lower debinding rate, performing debinding in a vacuum atmosphere, and changing the particle size inside the filament.

Despite the progress made by previous studies using the aforementioned solutions, challenges related to time and equipment requirements remain. The primary drawback of thermal debinding is its long production cycle, which is prolonged by a long holding time and low heating rate. Temperature plays a dominant role in the thermal debinding process. However, to the best of our knowledge, only a few studies have been conducted on heat transfer processes during thermal debinding, such as heat exchange and temperature transfer inside the kiln and crucible. Over the past two decades, numerous experiments have been carried out using computational fluid dynamics (CFD) simulations to analyze the oven heating process [[21], [22], [23], [24], [25], [26], [27], [28]], which could be an excellent method to study the temperature field during debinding. However, due to computing capacity and efficiency limitations in the studies mentioned above, baking ovens were only simulated at heating temperatures of 100 K–500 K [21,[23], [24], [25], [26], [27],^29^], which are lower than the debinding temperature of metals. The heat transfer mechanism in the thermal debinding process is still unclear. Further studies using CFD modeling are required to investigate temperature changes and optimize distortion during the thermal debinding process. Additionally, there is an urgent need for a novel and generic method to control porosity in the manufacturing industry.

**Fig. 1:**
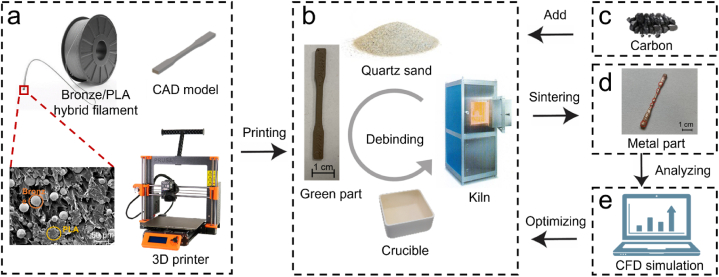
Schematic diagram of the optimized process: (a) 3D printing; (b) Thermal debinding process; (c) Adding carbon to thermal sintering process; (d) Producing metal part; (e) Analyzing and optimizing using CFD.

In this study, we developed a combined CFD simulation and experimental method to optimize the thermal debinding process and the distortion of metal parts. The schematic diagram of the proposed model is shown in Fig. 1. The green parts were printed (Fig. 1(a)) and placed inside an alumina crucible surrounded by quartz sand for thermal debinding (Fig. 1 (b)). From Fig. 1 (c) to Fig. 1 (d), the brown parts were coated with carbon and sintered to produce the metal parts. Finally, in Fig. 1 (e), we used the defects in the produced metal parts and debinding conditions to simulate and analyze the changes and distribution of heat inside the kiln and differently sized crucibles during debinding. We evaluated the quality of the metal parts using porosity and tensile strength as the main indices to determine whether they met the required standards. For metal parts with poor quality, we adjusted the experimental parameters in the debinding process. The conclusions in this paper can promote the application of MEX-produced metal parts in industrial fields.

## 2 Materials and methods

### 2.1 Geometry configuration

**Fig. 2:**
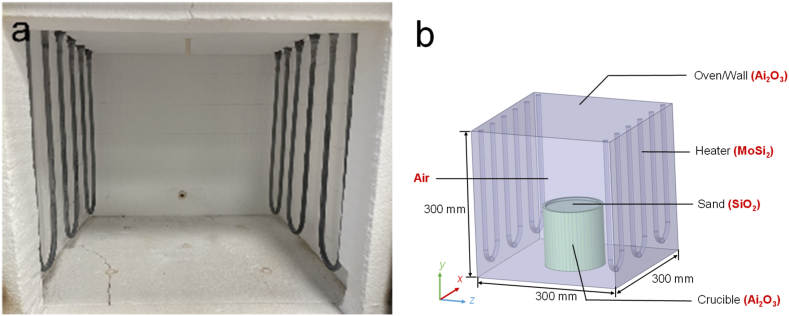
Kiln Geometry: (a) Experimental kiln; (b) Kiln components and materials used in the simulation.

We utilized a Quis 20 sintering kiln (Thermo-Star GmbH, Aachen, Germany) for debinding and sintering metal parts. The simulation geometry was constructed based on that of the kiln depicted in Fig. 2(a). The total kiln capacity is 27 L, and the chamber's width, depth, and height measure 300 mm, as illustrated in Fig. 2(b). The kiln features six U-shaped heaters composed of molybdenum disilicide, and the six walls (including the kiln door) are constructed of aluminum oxide fiber insulation. To effectively prevent air from entering the crucible during sintering, we utilized alumina crucibles, while quartz sand was used in the absence of a fan, seal equipment, or inert gas input in the kiln. As a result, thermal processes take place in an open atmosphere. Table 1 [[30], [31], [32], [33], [34], [35]] displays the physical properties of the constituent materials.

**Table 1:** Physical properties of the component materials.

Component	Fluid	Kiln/crucible	Sand	Heater
Material	Air	Aluminum oxide	Silicon oxide	Molybdenum disilicide
Density (kg/m^3^)	1.225	3987	2660	6260
Specific heat (J/(kg·K))	1005	718	74	437
Thermal conductivity (W/(m·K)	0.0262	36	3	66.2
Emissivity (%)	80	80	79	75

### 2.2 Numerical discretization

**Fig. 3:**
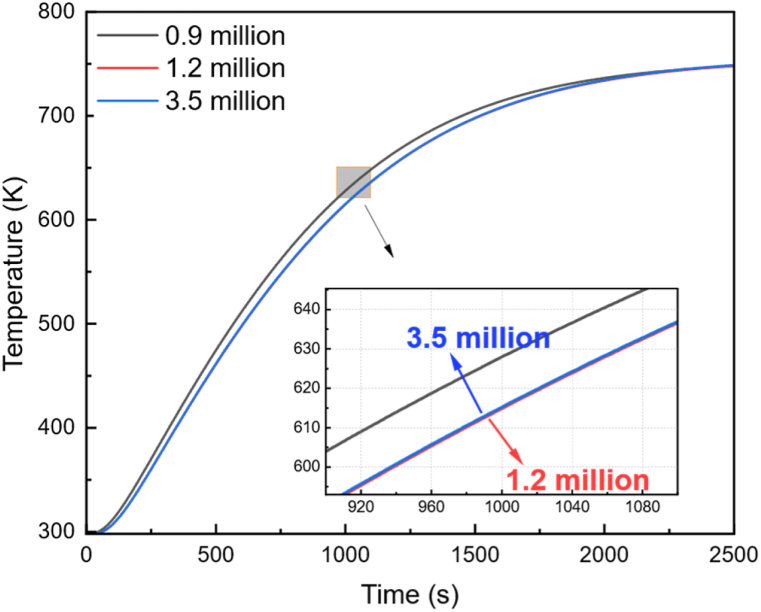
Comparison of simulation results using meshes with varying numbers of elements: 0.9 million (black curve), 1.2 million (red curve), and 3.5 million (blue curve). The blue and red curves almost coincide. (For interpretation of the references to color in this figure legend, the reader is referred to the Web version of this article.)

The CFD simulation was conducted utilizing ANSYS-FLUENT (ANSYS Inc, Canonsburg, USA) software. A high-quality mesh was employed to discretize the generated geometrical model. In the initial stage, mesh independence was verified by conducting preliminary computations using three meshes with gradually increasing numbers of elements. Meshes consisting of 0.9 million, 1.2 million, and 3.5 million elements were utilized to simulate the fundamental configuration of the kiln, as depicted in Fig. 3. The black curve was produced utilizing 0.9 million elements, and there was minimal difference between the red curve generated using 1.2 million elements and the blue curve using 3.5 million elements, which essentially overlapped. Based on these results, the grid with 1.2 million mesh elements was selected for further analysis. Most of the small mesh elements were implemented to model the U-shaped heaters and crucible. The minimum grid size was 0.1 mm, and the maximum grid size was 0.9 mm. All simulations were performed on a workstation with 8 cores and 64 GB memory, with a computational time of 2–3 days.

### 2.3 Simulation details

**Fig. 4:**
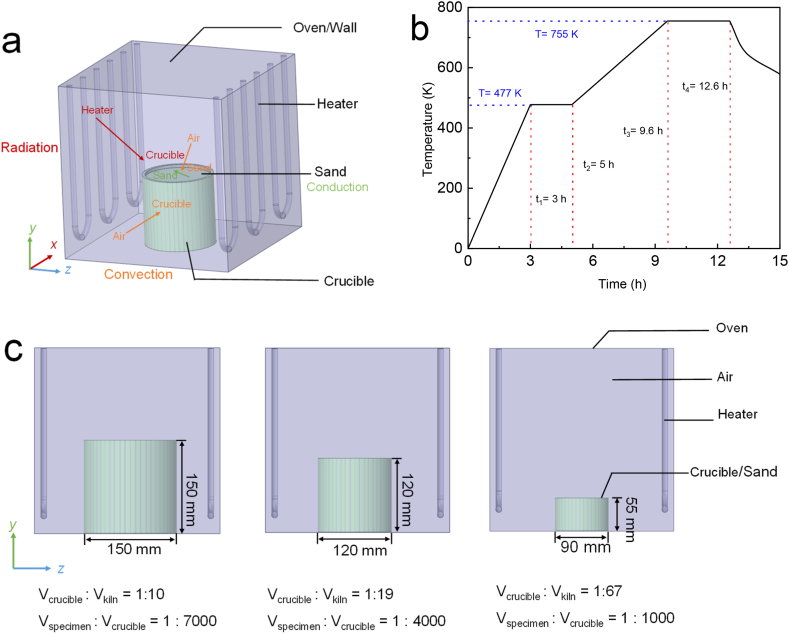
Heating setup for simulation: (a) A diagram illustrating the energy transfer process in the kiln; (b) The original heat treatment program used in the thermal debinding process experiments. After *t*_*3*_ represents the simulated temperature in the kiln; (C) Crucible-2.7; Crucible-1.4; and Crucible-0.4.

The heat transfer process during the transient operation of the device includes radiation, conduction and convection [22,27], as depicted in Fig. 4 (a). Radiation is the primary mode of heat transfer in a kiln, with the heater serving as the source of radiant heat in the system. Heat is conducted inside the device through the air, kiln walls, the crucible, and sand. Given these conditions, we used the discrete ordinate (DO) model to describe the radiation process [21,26,27,36,37]. We adopted the Rayleigh-Benard-free convection model to describe the convection process in the air, as the kiln does not contain a fan or air outlet. Free convection through the air typically has only a minor effect on the sintering kiln. The heat transfer coefficient of air ranges between 5 and 25 W/(m^2^·K). Modeling the geometry and various components of the kiln, as well as the enormous simulation time, increases computational effort to an undesirable level. Hence, we chose the laminar model to describe fluid viscosity, given the computing capacity based on the turbulence model and the slight influence on the temperature field [21,22,38,39].

Mixed convective and radiative boundary conditions were employed for the six walls of the kiln, and convection, conduction, and radiation processes were simulated for each component of the kiln. During debinding, the kiln was heated to 477 K for 3 h and held at this temperature for 2 h, followed by heating for 4.6 h to 755 K (1 K/min) and holding at this temperature for 3 h (Fig. 4 (b)). In this simulation, the heat treatment program was simplified from that shown after *t*_*3*_ in Fig. 4 (b). The same heating process was used for different crucibles, and the sand was held at a debinding temperature of 755 K for 7000 s. At the end of the temperature holding time, the heaters were turned off, and the system was allowed to cool for 2000 s. The initial temperature of the heaters was set to 755 K, while the air, crucible, sand, and kiln were initialized to a room temperature of 298 K.

In this study, we modeled three different sizes of crucibles, as shown in Fig. 4 (c) a large crucible with a volume of 2.7 L (Crucible-2.7), a medium crucible with a volume of 1.4 L (Crucible-1.4), and a small crucible with a volume of 0.4 L (Crucible-0.4). The temperature transformation in the center of the crucible/sand was analyzed to explore the temperature effect on debinding, and the differences between the three crucibles were compared and discussed. Additionally, we investigated the heat difference in Crucible-2.7.

### 2.4 Materials and specimen manufacturing

We used a bronze/polylactide (PLA) composite filament (The Virtual Foundry, Inc, Stoughton, USA) to manufacture metal tensile specimens. The filament used in this work contains 15 wt% PLA, 85 wt% bronze powder and trace synthetic material/binding additive. The tensile specimens, with a 2.0-mm thickness, a 50.0-mm overall length and a 10.2-mm measure length, were fabricated based on DIN EN ISO 527-2: 2012-06, type 1BB [40,41].

The tensile specimens were printed by a desktop 3D printer Prusa i3 MK3 (Prusa Research a.s., Prague, Czech Republic), corresponding to the same process as regular PLA MEX 3D printing. Considering the hardness of the bronze particles, a steel extrusion nozzle was chosen for the 3D printer to prevent extrusion nozzle abrasion. To enhance the flow from the extrusion nozzle to the build platform, an extrusion nozzle diameter of 0.6 mm was selected, and the thickness of the layer reached 0.3 mm.

Debinding and sintering took place during the thermal processes. The printed parts were positioned inside an alumina crucible along with quartz sand and subjected to heat treatment as illustrated in Fig. 4 (b), following standard procedures for debinding. At the end of the debinding holding time, heating was stopped, and the system was allowed to cool down. The quartz sand was covered with superfluous carbon during the sintering process to prevent metal oxidation at high temperatures. The brown parts were heated to a sintering temperature of 1144 K at a low rate and held at this temperature for 3 h.

We determine the temperature changes in the system by using different-sized crucibles to experimentally fabricate metal specimens, as shown in Fig. 4 (c). As PLA has a vaporization point of 633 K, the PLA binder was vaporized from 477 K to 755 K during the second step of the debinding process, as shown in Fig. 4 (b). We explored the effects of the heat treatment program on the metal parts by reducing the heating rate and extending the debinding holding time using Crucible-0.4. The experimental details are shown in Table 2.

**Table 2:** Experimental details with three different sizes of crucibles and the heat treatment program.

No.	Crucible	Heating rate (K/min)	Holding time (h)
1	Crucible-0.4	1	3
2	Crucible-1.4	1	3
3	Crucible-2.7	1	3
4	Crucible-0.4	0.5	3
5	Crucible-0.4	1	6
6	Crucible-0.4	0.5	6

### 2.5 Microstructural characterization

Microstructural analysis of the specimens was conducted, and images of the metal's microstructure and pores were captured. Cross-sections of the fabricated metal specimens were ground, polished, and visualized using a digital microscope, the KEYENCE VHX-5000, from Keyence Corporation of America located in Elmwood Park, NJ, USA. To construct the cross-section images and quantify specimen porosity, we utilized the ZEISS image analyzer software from Carl ZEISS Microscopy GmbH of Jena, Germany.

### 2.6 Mechanical characterization

The sintered components displayed an approximately 20% reduction in the *x* and *y* dimensions, whereas the shrinkage in the *z* dimension was lower due to the anisotropic nature of the specimens [4]. To examine the tensile properties of the specimens, we employed a universal testing machine, the TT28100, from TIRAtest GmbH in Schalkau, Germany. The tensile test was carried out according to DIN EN ISO 6892–1:2020-6, with a traverse travel rate of 1 mm/min. The strain-tensile stress curve acquired during the tensile test was recorded and analyzed.

## 3 Results and discussion

### 3.1 Analysis of the temperature field in the kiln

This section presents and examines the temperature distribution within the kiln. The simulation outcomes and analyses are detailed in Sections 3.1, 3.2, while Sections 3.3, 3.4 present the validation of these results against experimental data. Each experimental group comprised ten parallel trials, and the average measurements were employed for analysis.

#### 3.1.1 Analysis of the heating process

**Fig. 5:**
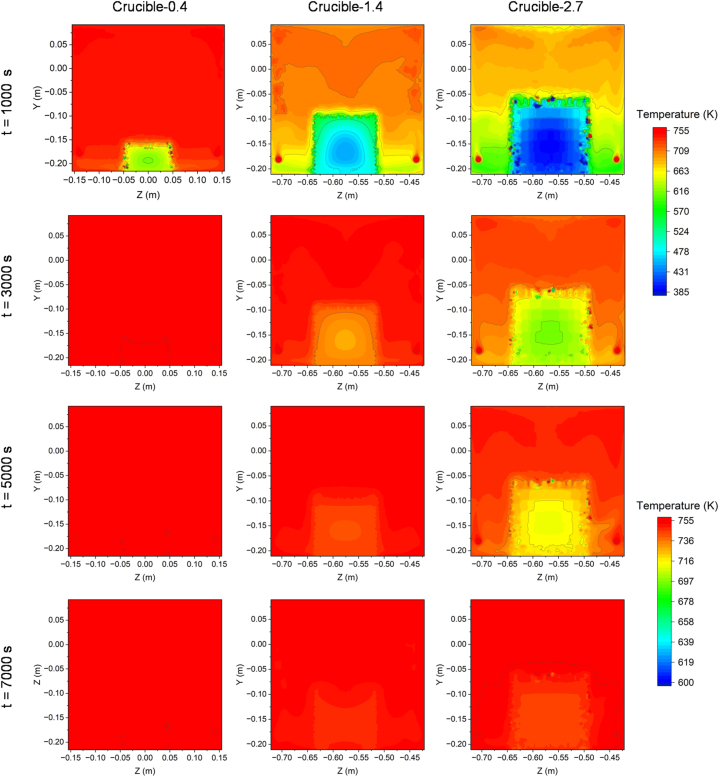
The time-dependent temperature distribution of three different-sized crucibles in the kiln during the heating process.

Fig. 5 shows the heat distribution of the *yz* cross-section of the specimens produced using the three differently sized crucibles during the heating process at 1000 s, 3000 s, 5000 s and 7000 s. The crucibles containing sand were placed in the middle of the bottom area of the kiln, and the circles at the left and right of the base correspond to the heater cross-sections. The remainder of the kiln volume was filled with air.

In Fig. 5, it can be observed that Crucible-0.4 underwent rapid heating to reach 755 K within approximately 3000 s. The entire system was held at this temperature for 7000 s. It is noteworthy that the heat transfer in Crucible-2.7 was slower, as evidenced by the gradual and continuous increase in temperature at the center of the crucible. Additionally, there were minor deviations between the final temperature and the target temperature of 755 K at 7000 s for Crucible-2.7. The temperature of Crucible-1.4 was found to be intermediate between those of Crucible-0.4 and Crucible-2.7. Overall, the temperature profiles reveal that smaller crucibles within the kiln can be heated more rapidly. However, it is important to note that the interior of the crucible was heated at a slower rate than the surrounding air.

**Fig. 6:**
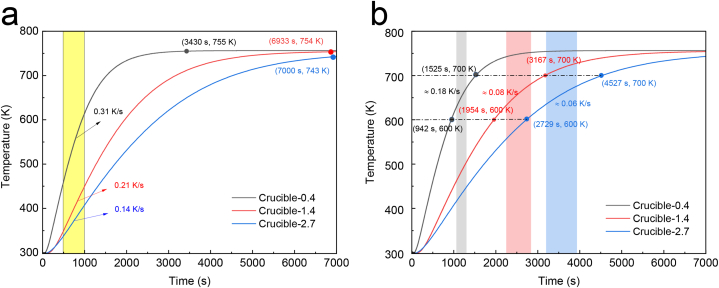
Analysis of the heating rate at the center of three differently sized crucibles: (a) Initial heating rate for the three crucibles from 500 s to 1000 s; (b) Heating rate for the three crucibles between 600 K and 700 K.

Fig. 6 illustrates the temperature changes and heating rates for three crucibles, as well as the trends for their center temperatures. The black curve (Crucible-0.4), red curve (Crucible-1.4), and blue curve (Crucible-2.7) depict the temperature profiles. The diagrams reveal that the temperature increased rapidly at first and then slowed down. In Fig. 6 (a), the initial heating rate between 500 s and 1000 s is displayed. The heating rates for Crucible-0.4, Crucible-1.4, and Crucible-2.7 were 0.31 K/s, 0.21 K/s, and 0.14 K/s, respectively. The heating rate of Crucible-0.4 was 1.5 times greater than that of Crucible-1.4 and 2.2 times greater than that of Crucible-2.7. Fig. 6 (b) presents the heating rates for the three curves between 600 K and 700 K, corresponding to the second step of the debinding process that is significant for PLA vaporization. The center was heated at a rate of 0.18 K/s for Crucible-0.4, 0.08 K/s for Crucible-1.4, and 0.06 K/s for Crucible-2.7. The heating rate of Crucible-0.4 was 2.3 times higher than that of Crucible-1.4 and three times higher than that of Crucible-2.7.

**Fig. 7:**
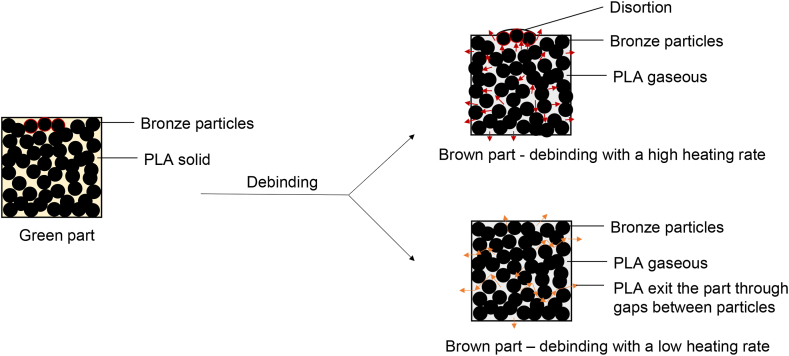
Thermal debinding process. A high heating rate created distortion. A low heating rate maintained the geometry.

During the debinding process, as shown in Fig. 7, the PLA binder vaporized and was removed from the green part as the temperature increased. It was crucial to prevent the bronze particles from moving to maintain the original geometry. However, under high heating rates, PLA vaporized at a faster rate than the gaseous PLA could escape from the green parts, generating immense pressure that pushed the bronze particles out of their original form. This resulted in the formation of pores inside the brown part and distortions caused by the moving particles, as seen in the three particles at the top of the green part in Fig. 7. Nevertheless, decreasing the heating rate slowed down the PLA vaporization rate, allowing the gaseous PLA to exit the green part through the gaps between the metal particles without exerting pressure on them. As a result, the geometry of the specimen was maintained during the thermal debinding process. As discussed in Section 1, many researchers have observed that decreasing the heating rate during the thermal debinding process results in metal parts with lower distortion/porosity, but it prolongs the heat treatment program and extends the production cycle. However, compared to the original heat treatment program, the heating rate during debinding was decreased by a factor of 2.3 for Crucible-1.4 and three times for Crucible-2.7.

#### 3.1.2 Analysis of the cooling process

**Fig. 8:**
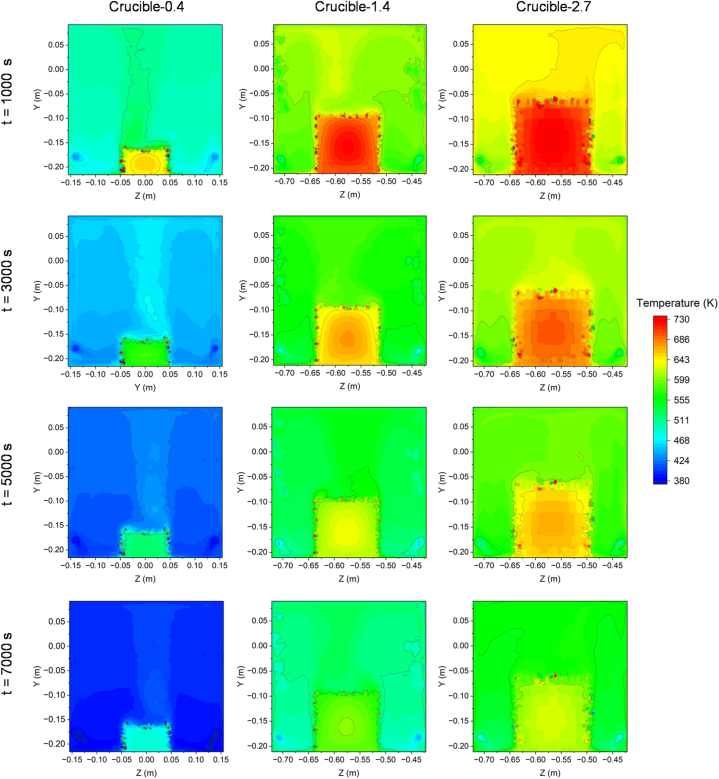
The time-dependent temperature distribution of three different-sized crucibles in the kiln during the cooling process.

Fig. 8 illustrates the cooling process of the three kilns at 500 s, 1000 s, 1500 s, and 2000 s. After being heated for 7000 s, the three kilns were allowed to cool down freely. The results indicate that Crucible-0.4 cooled down faster than Crucible-1.4 and Crucible-2.7. During the first 500 s, Crucible-1.4 and Crucible-2.7 had high temperatures, while Crucible-0.4 had already cooled down to 650 K. The color changes in Fig. 8 represent the temperature variation of the entire system.

**Fig. 9:**
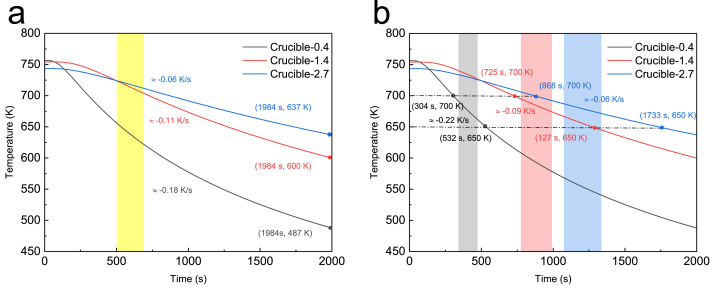
Analysis of the cooling rate at the center point of three crucibles of varying sizes is presented: (a) the cooling rate of the three crucibles during the initial cooling process from 500 s to 700 s, and (b) the cooling rate of the three crucibles between 700 K and 650 K. The data is reported in accordance with standard scientific notation and conventions.

Fig. 9 illustrates the cooling rates at the centers of the three crucibles during the free cooling process at 2000 s. Fig. 9 (a) displays the cooling rates at the beginning of the process, from 500 to 700 s. The cooling rates were −0.18 K/s for Crucible-0.4, −0.11 K/s for Crucible-1.4, and −0.06 K/s for Crucible-2.7. Crucible-0.4 cooled 1.6 times faster than Crucible-1.4 and three times faster than Crucible-2.7. Fig. 9 (b) displays the cooling rates of the three crucible centers between 700 K and 650 K. The temperature changed at a cooling rate of −0.22 K/s for Crucible-0.4, −0.09 K/s for Crucible-1.4, and −0.06 K/s for Crucible-2.7. During the cooling process from 700 K to 650 K, Crucible-0.4 cooled 2.4 times more slowly than Crucible-1.4 and 3.6 times than Crucible-2.7. The thermal debinding process was completed upon cooling the crucibles to 650 K, and there was no further vaporization of PLA. The results indicate that Crucible-2.7 and Crucible-1.4 retained heat longer than Crucible-0.4 and cooled considerably more slowly than Crucible-0.4.

The heating and cooling rates of Crucible-2.7 were considerably lower than those of the other crucibles. This low heating rate retarded the vaporization of PLA and minimized distortion. In comparison, Crucible-1.4 and Crucible-2.7 had a lower cooling rate and retained more heat, resulting in a slower decrease in the temperature of the crucible and sand. However, a lower cooling rate corresponds to a longer total reaction time and must be selected based on the product requirements in practice. In summary, the experimental results indicated that the debinding process was more efficient for Crucible-1.4 and Crucible-2.7 than for Crucible-0.4, without changing the heat treatment program and extending the heating cycle, which facilitates the production of high-quality metal parts.

### 3.2 Analysis of the temperature field in the crucible interior

**Fig. 10:**
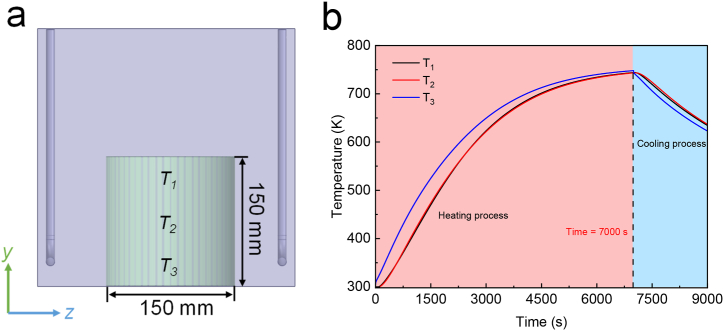
Temperature transfer at different positions within Crucible-2.7 during the heating and cooling processes: (a) Positions of three measurement points; (b) Temperature results at the three measurement points.

The temperatures at different positions within the crucible/sand system were found to be heterogeneous. We measured temperatures at three positions, namely the top (*T*_*1*_), middle (*T*_*2*_), and bottom (*T*_*3*_), in Crucible-2.7 (Fig. 10 (a)) and analyzed the temperature evolution during the heat treatment program. The results are presented in Fig. 10 (b). The temperature at the top position (blue curve) exhibited a faster heating and cooling rate compared to the middle and bottom positions (red and black curves). This could be attributed to the position of the heaters, which were located parallel and above the crucible. Consequently, the top position received radiation before the bottom part. Additionally, the energy and heated air had low density and flowed upward, causing rapid heat loss at the top position during the initial cooling phase. Interestingly, the temperature profiles of the middle and bottom positions showed a similar slower heating and cooling rate than the top position. Thus, embedding brown parts in the lower half of the crucible could potentially yield metal parts of higher quality.

### 3.3 Analysis of porosity and microstructure

**Fig. 11:**
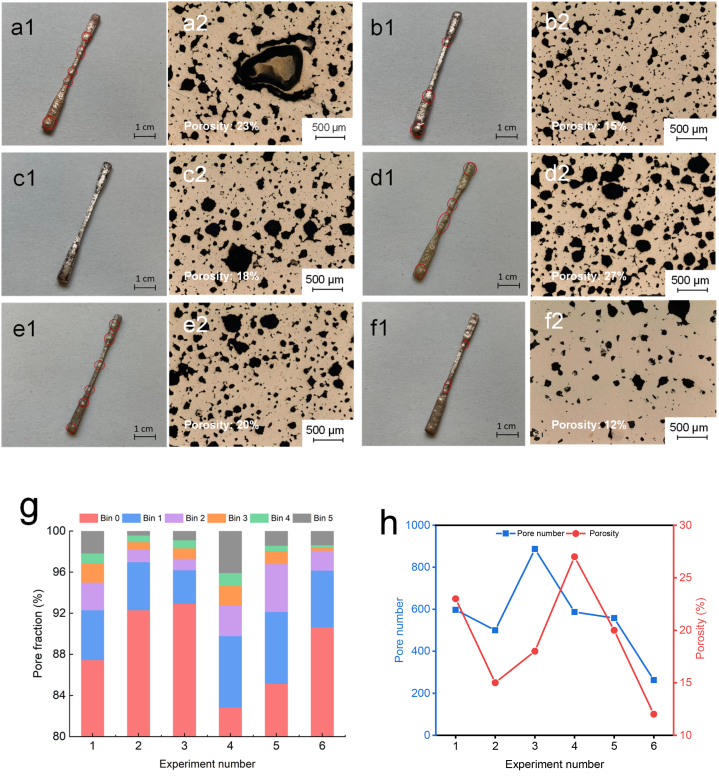
Analysis of Porosity and Microstructure: (a1-f1) Surface pores in sintered metal specimens from Experiments 1 to 6, as shown in Table 2, Section 2.4; (a2-f2) Microstructure of the six specimens from Experiments 1 to 6, as shown in Table 2, Section 2.4; (g) The pore fraction of each experiment; (h) Comparation of Pore number and porosity between Experiments No. 1 to 6.

Fig. 11 (a) to (f) illustrate the outcomes of Experiments No. 1 to 6 (as presented in Table 2, Section 2.4) and the porosity analysis. The metal products enumerated in Table 2 are depicted in Fig. 11 (a1) to (f1), and the corresponding microstructures are portrayed in Fig. 11 (a2) to (f2). Samples (a), (b), and (c) represent metal products fabricated in Crucible-0.4, Crucible-1.4, and Crucible-2.7, respectively, by employing the original heat treatment program. The results indicate that most pores were generated using Crucible-0.4, and numerous pores were observed on the surface of the metal part created with Crucible-0.4. By contrast, the metal parts produced using Crucible-1.4 and Crucible-2.7 exhibited a lesser number of pores. However, the tensile specimen produced in Crucible-2.7 was longer and thinner than the ones fabricated in Crucible-0.4 and Crucible-1.4, and the metal part generated in Crucible-2.7 was less substantial. Furthermore, the microstructure results (a2) revealed that the metal part interior had large pores and high porosity (23%) due to surface distortion of the metal part. The microstructure findings showed that the porosity of the metal part created in Crucible-2.7 (c2) was 18% higher than that of the metal part produced in Crucible-1.4, which had the best microstructure with a porosity of 15% (b2).

The metal specimens produced using Crucible-0.4 with a lower heating rate and longer holding time are presented in (d) to (f). The cross-sections of the metal parts displayed in (d2) to (e2) did not exhibit any significant large pores, but the distortion and porosity remained high. The microstructure of the specimens in (d) and (e) suggested less improvement in surface quality and porosity reduction than expected. Lowering only the heating rate or extending the holding time for debinding using Crucible-0.4 did not result in a significant improvement. However, the specimen shown in (f), which was prepared using a combination of the heating conditions used for specimens (d) and (e) - a low heating rate and long holding time - yielded better results, resulting in lower distortion and porosity of the interior of the resulting metal part. The disadvantage of these conditions is that they would extend the production period.

Fig. 11 (g) presents the percentage composition of pore size for Experiments 1 to 6. We utilized a MATLAB program to analyze the cross-sectional microstructure images of each specimen, which enabled us to determine the number of pores and the size of each pore. The pore size was categorized into six groups: bin 0 to bin 5, which corresponds to pores with areas ranging from 0 to 100, 100–200, 200–300, 300–400, 400–500, and greater than 500 square microns, respectively. As depicted in Fig. 11 (g), experiments 1, 4, 5, and 6 exhibit a certain proportion of large pores (bin 5), which is also manifested in the surface distortion of the samples. The extent of surface distortion is directly proportional to the size of the internal pores. A preponderance of large pores in the metal specimens renders them vulnerable to fractures where these pores are present. Conversely, experiments 2 and 3 predominantly contain small pores. Fig. 11 (h) presents a comparison of the total number and porosity of pores in the six experiments. Experiments 2 and 6 exhibited the lowest porosity, meaning that they possess the lowest total pore area. Consequently, these experiments exhibited a denser metal texture, leading to greater density and stronger mechanical properties.

### 3.4 Analysis of tensile test results

**Fig. 12:**
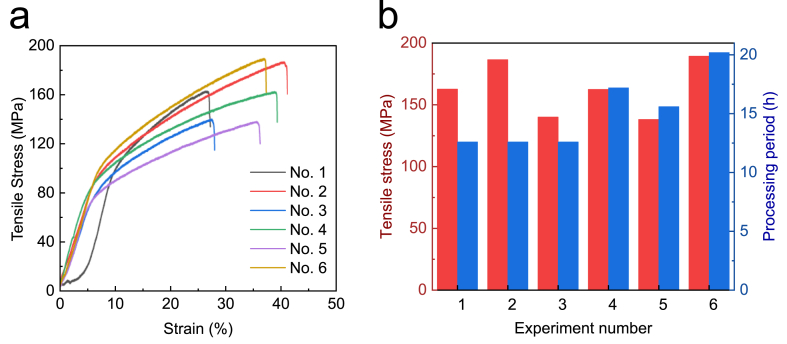
Analysis of Tensile Testing: (a) Tensile stress-strain curves of the metal parts obtained from Experiments No. 1 to 6 as listed in Table 2, Section 2.4; (b) Comparison between the tensile stress and processing period of debinding for Experiments No. 1 to 6.

Fig. 12 (a) displays the stress-strain curve of the tensile test results obtained from Experiments No. 1 to No. 6 (refer to Table 2). The tensile stress recorded in Experiment 2 was 186 MPa, which was superior to the results obtained from Experiments No. 1 and No. 3 performed under the original heat treatment conditions. However, reducing the heating rate (No. 4) and extending the holding time (No. 5) using Crucible-0.4 did not result in a significant improvement in the tensile stress of the prepared specimen. Nonetheless, the tensile stress of 189 MPa recorded in No. 6 was excellent compared to those measured in No. 4 and No. 5 and identical to that measured in No. 2, which used Crucible-1.4.

Fig. 12 (b) depicts the tensile stress and processing period of thermal debinding for Experiments No. 1 to No. 6. The tensile stress of No. 2, prepared using Crucible-1.4, was similar to that of No. 6, prepared using Crucible-0.4. Increasing the period of the debinding process can enhance the mechanical properties of metal parts. However, the longest debinding process was conducted in Experiment No. 6, which totaled 20.2 h and is not cost-effective. In contrast, the metal parts produced in Crucible-1.4 using the original heat treatment exhibited a significant increase in tensile stress, from 138 MPa to 186 MPa, with only a 12.6-h debinding process, representing a reduction of 37.6% in processing time.

The simulation results provide a theoretical basis for improving the experimental methods and enhancing product quality. Increasing the volume of the sand and crucible (from 0.4 L to 1.4 L) decreased the heating rate for the same heat treatment program, which resulted in better metal parts with fewer pores. In this study, the optimal volume ratio corresponded to a crucible-to-kiln volume ratio of 1:19 and a specimen-to-crucible volume ratio of 1:4000. The heating rate was slowed down during the heating process before *t*_*3*_ (see Fig. 4 (b)). Moreover, increasing the volume of the sand and crucible slowed down the cooling rate (after *t*_*4*_), which gave gaseous PLA more time to escape from the green parts. However, an excessively large volume of the crucible or sand prevented the temperature at the crucible center from reaching the target debinding temperature or sintering temperature, leading to inadequate sintering for the same heat program. To achieve a short production process while reducing the distortion and tensile stress of metal parts, Crucible-1.4 was found to be the best choice.

## 4 Conclusions

In this study, we conducted experimental tests and numerical simulations to optimize the distortion of the metal parts in the thermal debinding-sintering process. The result showed that the small crucible (crucible: kiln = 1:67) caused porosity and distortion of the parts due to excessive PLA evaporation in thermal debinding process. Lowering the heating rate and increasing the holding time mitigated these defects but prolonged the processing time. The large crucible (crucible: kiln = 1:10) failed to reach the target temperature due to its low heating rate, negatively impacting thermal debinding and sintering and resulting in weak metal parts with poor tensile strength. In contrast, the medium crucible (crucible: kiln = 1:19) decreased the porosity of the metal parts from 27% to 15% without extending processing time, and increased the tensile strength of the parts from 138 MPa to 186 MPa. Thermal debinding with the medium crucible proved to be most efficient and reduced processing time by 37.6% when compared to the small crucible.

In numerical simulations, we obtained temperature field data during the debinding process for the kiln with various crucibles. The heating rate of the specimen was measured at the central position of the crucibles. The metal parts in the small crucible experienced notably quicker heating compared to those in the medium and large crucibles, which agreed favorably with the experimental results. This explained the excessive evaporation of PLA in the small crucible during the debinding process.

Despite the improved quality of metal parts when utilizing the medium crucible, porosities remained above 10%. Future efforts should focus on further reducing the porosity of metal parts to below 5%. Nonetheless, the method employed, which combines experimental tests and numerical simulations, demonstrated significant potential in enhancing the distortion control of metal parts during the thermal debinding-sintering process.

## Data availability statement

The authors confirm that the data that support the findings of this study are available from the corresponding author upon reasonable request.

## CRediT authorship contribution statement

**Xueying Wei:** Conceptualization, Data curation, Investigation, Methodology, Software, Validation, Visualization, Writing – original draft, Writing – review & editing. **Xujun Li:** Methodology, Software, Writing – review & editing. **Rüdiger Bähr:** Funding acquisition, Supervision, Writing – review & editing.

## Declaration of competing interest

The authors declare that they have no known competing financial interests or personal relationships that could have appeared to influence the work reported in this paper.

## References

[1] Contreras J.M., Jiménez-Morales A., Torralba J.M. (2009). Fabrication of bronze components by metal injection moulding using powders with different particle characteristics. J. Mater. Process. Technol..

[2] Xu Z., Hodgson M.A., Chang K., Chen G., Yuan X., Cao P. (2017). Effect of sintering time on the densification, microstructure, weight loss and tensile properties of a powder metallurgical Fe-Mn-Si alloy. Metals.

[3] Tafti A.A., Demers V., Majdi S.M., Vachon G., Brailovski V. (2021). Effect of thermal debinding conditions on the sintered density of low-pressure powder injection molded iron parts. Metals.

[4] Wei X., Behm I., Winkler T., Scharf S., Li X., Bähr R. (2022). Experimental study on metal parts under variable 3D printing and sintering orientations using bronze/PLA hybrid filament coupled with fused filament fabrication. Materials.

[5] Barriere T., Liu B., Gelin J.C. (2003). Determination of the optimal process parameters in metal injection molding from experiments and numerical modeling. J. Mater. Process. Technol..

[6] Ramazani H., Kami A. (2022). Metal FDM, a new extrusion-based additive manufacturing technology for manufacturing of metallic parts: a review. Prog. Appl. Manuf..

[7] Tosto C., Tirillò J., Sarasini F., Cicala G. (2021). Hybrid metal/polymer filaments for fused filament fabrication (FFF) to print metal parts. Appl. Sci..

[8] Rosnitschek T., Glamsch J., Lange C., Alber-Laukant B., Rieg F. (2021). An automated open-source approach for debinding simulation in metal extrusion additive manufacturing. Designs.

[9] Agne A., Barrière T. (2017). Modelling and numerical simulation of supercritical CO2 debinding of inconel 718 components elaborated by metal-injection molding. Appl. Sci..

[10] Sadaf M., Bragaglia M., Nanni F. (2021). A simple route for additive manufacturing of 316L stainless steel via Fused Filament Fabrication. J. Manuf. Process..

[11] Gonzalez-Gutierrez J., Cano S., Schuschnigg S., Kukla C., Sapkota J., Holzer C. (2018). Additive manufacturing of metallic and ceramic components by the material extrusion of highly-filled polymers: a review and future perspectives. Materials.

[12] Hasib A.G., Niauzorau S., Xu W., Niverty S., Kublik N., Williams J., Chawla N., Song K., Azeredo B. (2021). Rheology scaling of spherical metal powders dispersed in thermoplastics and its correlation to the extrudability of filaments for 3D printing. Addit. Manuf..

[13] Singh G., Missiaen J.-M., Bouvard D., Chaix J.-M. (2021). Copper additive manufacturing using MIM feedstock: adjustment of printing, debinding, and sintering parameters for processing dense and defectless parts. Int. J. Adv. Manuf. Technol..

[14] Amin A., Ibrahim M., Asmawi R., Mustaffa N., Hashim M. (2017). IOP Conference Series: Materials Science and Engineering.

[15] Hwang K.S., Tsou T.H. (1992). Thermal debinding of powder injection molded parts: observations and mechanisms. Metall. Trans. A.

[16] Supriadi S., Suharno B., Hidayatullah R., Maulana G., Baek E.R. (2017). Thermal debinding process of SS 17-4 PH in metal injection molding process with variation of heating rates, temperatures, and holding times. Solid State Phenom..

[17] Mousapour M., Salmi M., Klemettinen L., Partanen J. (2021). Feasibility study of producing multi-metal parts by Fused Filament Fabrication (FFF) technique. J. Manuf. Process..

[18] Enneti R.K., Park S.J., German R.M., Atre S.V. (2012). Review: thermal debinding process in particulate materials processing. Mater. Manuf. Processes.

[19] Thompson Y., Gonzalez-Gutierrez J., Kukla C., Felfer P. (2019). Fused filament fabrication, debinding and sintering as a low cost additive manufacturing method of 316L stainless steel. Addit. Manuf..

[20] Singh P., Balla V.K., Gokce A., Atre S.V., Kate K.H. (2021). Additive manufacturing of Ti-6Al-4V alloy by metal fused filament fabrication (MF3): producing parts comparable to that of metal injection molding. Prog. Appl. Manuf..

[21] Chhanwal N., Tank A., Raghavarao K.S.M.S., Anandharamakrishnan C. (2012). Computational fluid dynamics (CFD) modeling for bread baking process—a review. Food Bioproc. Tech..

[22] Fu Z., Yu X., Shang H., Wang Z., Zhang Z. (2019). A new modelling method for superalloy heating in resistance furnace using FLUENT. Int. J. Heat Mass Tran..

[23] Smolka J., Bulinski Z., Nowak A.J. (2013). The experimental validation of a CFD model for a heating oven with natural air circulation. Appl. Therm. Eng..

[24] Verboven P., Scheerlinck N., De Baerdemaeker J., Nicolaï B.M. (2000). Computational fluid dynamics modelling and validation of the temperature distribution in a forced convection oven. J. Food Eng..

[25] Therdthai N., Zhou W., Adamczak T. (2004). Three-dimensional CFD modelling and simulation of the temperature profiles and airflow patterns during a continuous industrial baking process. J. Food Eng..

[26] Smolka J. (2013). Genetic algorithm shape optimisation of a natural air circulation heating oven based on an experimentally validated 3-D CFD model. Int. J. Therm. Sci..

[27] Rek Z., Rudolf M., Zun I. (2012). Application of CFD simulation in the development of a new generation heating oven. Stroj Vestin-J. Mech. Eng..

[28] Therdthai N., Zhou W., Adamczak T. (2003). Two-dimensional CFD modelling and simulation of an industrial continuous bread baking oven. J. Food Eng..

[29] Mirade P.S., Daudin J.D., Ducept F., Trystram G., Clément J. (2004). Characterization and CFD modelling of air temperature and velocity profiles in an industrial biscuit baking tunnel oven. Food Res. Int..

[30] Li B., Oliveira F.A.C., Rodríguez J., Fernandes J.C., Rosa L.G. (2015). Numerical and experimental study on improving temperature uniformity of solar furnaces for materials processing. Sol. Energy.

[31] Utomo A.T., Poth H., Robbins P.T., Pacek A.W. (2012). Experimental and theoretical studies of thermal conductivity, viscosity and heat transfer coefficient of titania and alumina nanofluids. Int. J. Heat Mass Tran..

[32] Teng Y.-Y., Chen J.-C., Lu C.-W., Chen H.-I., Hsu C., Chen C.-Y. (2011). Effects of the furnace pressure on oxygen and silicon oxide distributions during the growth of multicrystalline silicon ingots by the directional solidification process. J. Cryst. Growth.

[33] Coskun C., Oktay Z., Ilten N. (2009). A new approach for simplifying the calculation of flue gas specific heat and specific exergy value depending on fuel composition. Energy.

[34] Nazififard M., Nematollahi M., Jafarpur K., Suh K.Y. (2012). Numerical simulation of water-based alumina nanofluid in subchannel geometry. Sci. Technol. Nucl. Ins..

[35] Zhang K., Feng Y., Schwarz P., Wang Z., Cooksey M. (2013). Computational fluid dynamics (CFD) modeling of bubble dynamics in the aluminum smelting process. Ind. Eng. Chem. Res..

[36] Bohlooli Arkhazloo N., Bouissa Y., Bazdidi-Tehrani F., Jadidi M., Morin J.-B., Jahazi M. (2019). Experimental and unsteady CFD analyses of the heating process of large size forgings in a gas-fired furnace. Case Stud. Therm. Eng..

[37] Anishaparvin A., Chhanwal N., Indrani D., Raghavarao K., Anandharamakrishnan C. (2010). An investigation of bread‐baking process in a pilot‐scale electrical heating oven using computational fluid dynamics. J. Food Sci..

[38] Mistry H., Ganapathi s., Dey S., Bishnoi P., Castillo J.L. (2006). Modeling of transient natural convection heat transfer in electric ovens. Appl. Therm. Eng..

[39] Abraham J.P., Sparrow E.M. (2003). Three-dimensional laminar and turbulent natural convection in a continuously/discretely wall-heated enclosure containing a thermal load. Numer. Heat. Tran. A-Appl.

[40] Dehne L., Vila Babarro C., Saake B., Schwarz K.U. (2016). Influence of lignin source and esterification on properties of lignin-polyethylene blends. Ind. Crop. Prod..

[41] Bremer M., Janoschek L., Kaschta D., Schneider N., Wahl M. (2022). Influence of plastic recycling—a feasibility study for additive manufacturing using glycol modified polyethylene terephthalate (PETG). SN Appl. Sci..

